# Retrograde optogenetic characterization of the pontospinal module of the locus coeruleus with a canine adenoviral vector

**DOI:** 10.1016/j.brainres.2016.02.023

**Published:** 2016-06-15

**Authors:** Yong Li, Louise Hickey, Ray Perrins, Emilie Werlen, Amisha A. Patel, Stefan Hirschberg, Matt W. Jones, Sara Salinas, Eric J. Kremer, Anthony E. Pickering

**Affiliations:** a School of Physiology & Pharmacology, University of Bristol, Bristol BS8 1TD, UK; b Institut de Génétique Moléculaire de Montpellier, CNRS UMR 5535, Montpellier, France; c Université de Montpellier, Montpellier, France; d Department of Anaesthesia, University Hospitals Bristol, Bristol BS2 8HW, UK

**Keywords:** Noradrenaline, Locus coeruleus, Pontospinal, Optogenetics, Retrograde vector

## Abstract

Noradrenergic neurons of the brainstem extend projections throughout the neuraxis to modulate a wide range of processes including attention, arousal, autonomic control and sensory processing. A spinal projection from the locus coeruleus (LC) is thought to regulate nociceptive processing. To characterize and selectively manipulate the pontospinal noradrenergic neurons in rats, we implemented a retrograde targeting strategy using a canine adenoviral vector to express channelrhodopsin2 (CAV2-PRS-ChR2-mCherry). LC microinjection of CAV2-PRS-ChR2-mCherry produced selective, stable, transduction of noradrenergic neurons allowing reliable opto-activation *in vitro*. The ChR2-transduced LC neurons were opto-identifiable *in vivo* and functional control was demonstrated for >6 months by evoked sleep-wake transitions. Spinal injection of CAV2-PRS-ChR2-mCherry retrogradely transduced pontine noradrenergic neurons, predominantly in the LC but also in A5 and A7. A pontospinal LC (ps:LC) module was identifiable, with somata located more ventrally within the nucleus and with a discrete subset of projection targets. These ps:LC neurons had distinct electrophysiological properties with shorter action potentials and smaller afterhyperpolarizations compared to neurons located in the core of the LC. *In vivo* recordings of ps:LC neurons showed a lower spontaneous firing frequency than those in the core and they were all excited by noxious stimuli. Using this CAV2-based approach we have demonstrated the ability to retrogradely target, characterise and optogenetically manipulate a central noradrenergic circuit and show that the ps:LC module forms a discrete unit.

*This article is part of a Special Issue entitled SI: Noradrenergic System*.

## Highlights

• Validated a canine adenoviral vector to transduce noradrenergic neurons.
• Robust and efficient retrograde optogenetic targeting of locus coeruleus.
• Pontospinal neurons of the locus coeruleus form a discrete module.

## 1 Introduction

The noradrenergic (NAergic) innervation of the brain and the spinal cord arises from several clusters of neurons in the pons and medulla (Dahlstrom and Fuxe, 1964). The locus coeruleus (LC), the largest of these cell groups, extends axonal projections throughout the neuraxis (Aston-Jones, 2004, Samuels and Szabadi, 2008). As a consequence, it has been considered to be a global effector system causing brain-wide state changes. However, the LC is involved in a diverse range of functions including attention, memory, sleep-wake, autonomic control and modulation of sensory input. This range of roles of the LC raises the question of how discrete functional specificity can be achieved at particular targets. This is the subject of active investigation with contrasting viewpoints: for example recent elegant tracing studies have demonstrated widespread and divergent projections from the LC to the forebrain (Schwarz et al., 2015) yet other investigators have provided evidence for regional specificity in the organization of the LC projection neurones and their electrophysiology (Chandler et al., 2014). An example of the striking functional contrasts in the role of the LC is apparent in the comparison between the role of the LC in salience detection-improving resolution in cortical signal processing (Aston-Jones and Cohen, 2005, Berridge and Waterhouse, 2003, Sara and Bouret, 2012) contrasted with its role in endogenous analgesic circuits – projecting to the spinal cord to selectively suppress the onward transmission of sensory information (Jones and Gebhart, 1986, Millan, 2002, Pertovaara, 2006).

Changes in the activity of the descending NAergic pain control system have been implicated in the pathology of chronic pain-particularly in neuropathic sensitization (Hayashida et al., 2012, Jasmin et al., 2003, Rahman et al., 2008, Viisanen and Pertovaara, 2007). A spinal segmental deficit in NAergic control is seen after peripheral nerve injury allowing localized nociceptive sensitization (Hughes et al., 2013). However, intrathecal pharmacological blockade with α2-adrenoceptor antagonists shows the descending NAergic projection is still partially limiting sensitization (De Felice et al., 2011, Hughes et al., 2013, Xu et al., 1999). Conversely augmentation of the descending NAergic system with intrathecal NA re-uptake inhibitors reverses sensitization indicating the potential benefit from restoration (Hughes et al., 2015).

Given the importance of this descending NAergic projection in the regulation of nociception and in the pathology of neuropathic pain, we sought to develop a means to target this control pathway to delineate mechanisms with spatial and temporal precision. Recent optogenetic targeting approaches have allowed specific activation of the NAergic neurons of the LC (Carter et al., 2010, Vazey and Aston-Jones, 2014). However, because of the widespread projections of the LC and the emerging evidence for the heterogeneity of these neurons (Chandler et al., 2014, Howorth et al., 2009a), it is perhaps not surprising that direct activation of the LC using an optogenetic approach uncovered both pro- and anti-nociceptive effects-potentially reflecting actions on distinct subgroups of LC neurons (Hickey et al., 2014).

Retrograde targeting of subsets of neurons offers a means to differentiate the functional roles of neuronal subgroups on the basis of their anatomical projections. Vectors based on human adenovirus type 5 (hAdV-C5) with the catecholaminergic selective synthetic promoter (PRS, (Hwang et al., 2001)), have previously been used to retrogradely target the pontospinal NAergic neurons and chronically explore their role in the regulation of nociception (Howorth et al., 2009a, Howorth et al., 2009b). However hAdV-C5 vectors lose efficacy for retrograde transduction at high titres (Howorth et al., 2009a), so we explored a different strategy to maximize expression instead employing canine adenovirus type 2 (CAV2) vectors that preferentially transduce neurons, are readily taken up at axon termini and transported to the cell body (Hnasko et al., 2006, Kremer et al., 2000, Salinas et al., 2009), and lead to stable expression (Bru et al., 2010). We show that CAV2 vectors containing the PRS promoter allow efficient transduction of LC neurons (both direct and retrograde) enabling reliable opto-activation. This approach permitted the anatomical and electrophysiological characterization of LC neurons projecting to the spinal cord showing that they form a specialized discrete module.

## 2 Results

### 2.1 Efficacy of direct LC transduction with CAV2-PRS-ChR2-mCherry

**Fig. 1:**
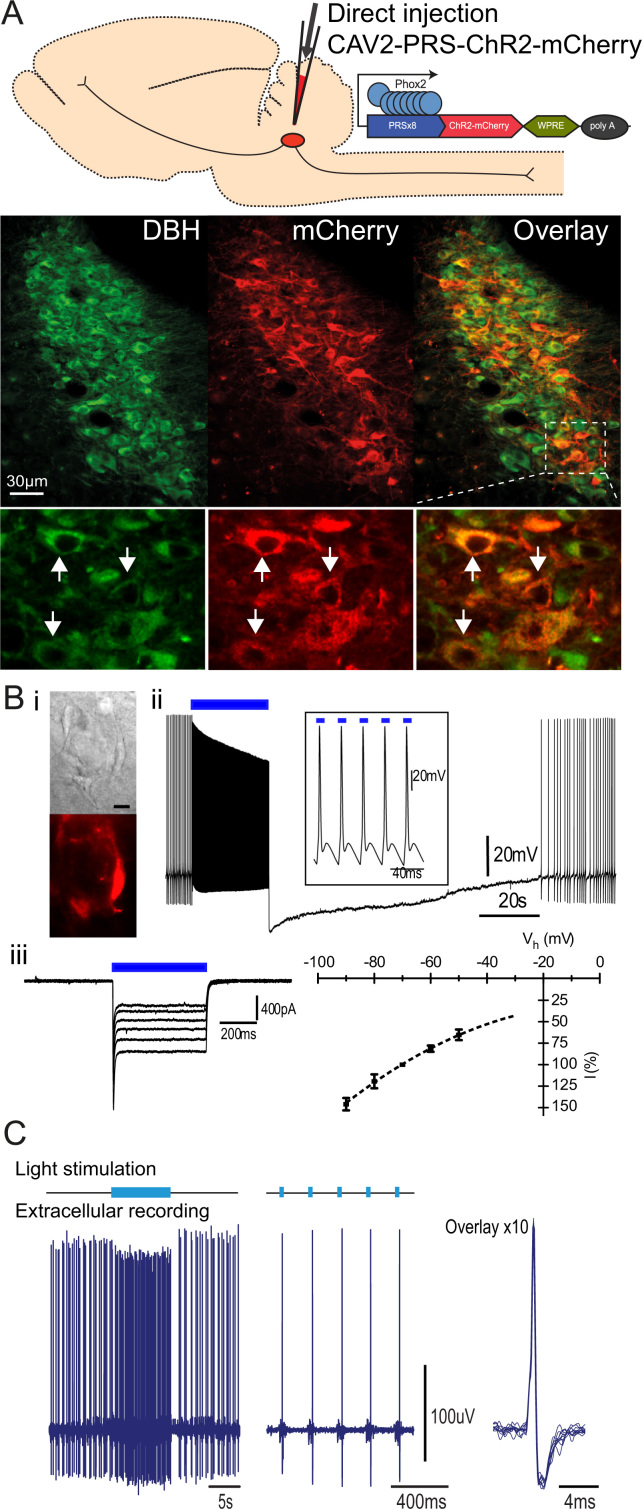
Selective, functional expression of ChR2-mCherry in the Locus Coeruleus. (A) Direct injection of CAV2-PRS-ChR2-mCherry efficiently transduced the LC neurons. Inset demonstrating co-localization of mCherry and DBH (1 µm confocal slice). (B) (i) Transduced LC neurons expressing ChR2-mCherry in acute pontine slices. (ii) Whole cell recording from LC neuron whose spontaneous firing is entrained by light pulses at 40 Hz (blue bar, 10 ms×10 mW, 473 nm, inset expanded). This high frequency evoked discharge is followed by a refractory period. (iii) Inward currents characteristic of ChR2 induced by light (500 ms×10 mW) at V_h_ −40 to −90 mV and plotted below as normalized steady state current (relative to *V*_h_ −70 mV, mean±SD, *n*=17). (C) Extracellular recording *in vivo* from a transduced LC neuron. Light pulses (473 nm; 15 mW×20 ms) entrained 1:1 neuronal firing at a frequency of 5 Hz (shown expanded on right with overlay of 10 spikes).

Direct injections of CAV2-PRS-ChR2-mCherry efficiently transduced the LC; fluorescence was restricted to dopamine β-hydroxylase (DBH)-immunoreactive somata (Fig. 1A, >98% of mCherry+ neurons double labeled) indicating that the selectivity for NAergic neurons of the PRS promoter (Hwang et al., 2001) is retained in this vector. Native mCherry-ChR2 fluorescence was seen in the somatic membrane within 7 days and expression remained stable for over 6 months post-injection. Given this pattern of transgene expression, whole cell patch clamp recordings were made *in vitro* 1–2 weeks post-transduction and behavioral/*in vivo* experiments commenced 3–4 weeks post-injection.

### 2.2 Optogenetic control of LC neurons using CAV2 vectors

Whole cell recordings of transduced LC neurons were made to determine the utility of the CAV2 vector for optogenetic studies. After direct LC injection of CAV2-PRS-ChR2-mCherry *in vivo,* there was strong fluorescent labeling of neurons in pontine slices (slices cut 7–14 days post injection). Whole cell recordings from mCherry+ LC neurons (Fig. 1Bi, *n*=24) showed light-evoked (*λ*=473 nm) action potential discharge, and trains of brief light pulses could drive one-for-one action potentials at up to 40 Hz (Fig. 1Bii). Following such bursts of driven discharge there was a prolonged refractory phase, typical of LC neurons (Cedarbaum and Aghajanian, 1978b). The light pulses elicited inward currents in voltage clamped neurons (Fig. 1Biii) that were characteristic of ChR2, with a rapidly inactivating component and a sustained steady state response. The steady state currents averaged 311±75pA (*n*=17, *V*_h_−60 mV, measured 200 ms after light onset) and showed an *I*–*V* relationship expected for ChR2 (non-selective cation conductance, Fig. 1Biii). All cells with mCherry fluorescence responded to light, while no fast inward current was seen in non-fluorescent LC neurons. These findings confirmed robust functional expression of ChR2 allowing optogenetic control of LC neurons.

Neurons transduced with CAV2-PRS-ChR2-mCherry showed the characteristic electrophysiological properties of the LC (Williams and Marshall, 1987). However, to detect any discrete changes in intrinsic properties following transduction their electrophysiological properties were compared with non-transduced LC neurons in the same slices and also to LC neurons of naïve rats. There was no significant difference between transduced versus non-transduced or naïve LC neurons for any of the intrinsic electrophysiological properties (Table 1). Prolonged periods of action potential discharge induced by light pulses (20–30 Hz for >1 min) did not affect the intrinsic neuronal properties and it was possible to repeatedly opto-stimulate the neurons at high frequencies for periods of over 1 h with no evidence of phototoxicity. Thus, neither CAV2 transduction, expression of ChR2 nor opto-activation produced any detrimental effects on LC neuronal properties.

### 2.3 Opto-identification of LC neurons *in vivo*

Extracellular recordings were made from LC neurons in anaesthetized rats to assess whether direct CAV2-PRS-ChR2-mCherry transduction would allow optogenetic control *in vivo*. Opto-activatable units (*n*=9) were identified in adult rats (*n*=5) at a depth of 5.3±0.1 mm from brain surface. Light activation of these LC neurons resulted in an immediate 3 to 4-fold increase in action potential discharge rate (single pulse increased firing from 3.7±0.8 to 12.8±3.6 Hz, *P*<0.05, *n*=8 neurons stimulated (1 s×20 mW), Fig. 1C). A majority of the LC neurons showed tight 1:1 spike coupling to short light pulses (5–100 ms, *n*=5/9, Fig. 1C), although the remaining neurons required a longer light pulse (~0.2–1 s). Presumably this reflects a variability in the level of intrinsic excitability of the recorded cell balanced against density of expression of ChR2 seen *in vivo* (see supplemental Fig. 1). The majority of identified LC neurons were noci-responsive showing an initial increase in firing to hindpaw pinch (5/6 cells tested).

### 2.4 LC transduction by CAV2 allows stable, reproducible opto-assay of behavior

**Fig. 2:**
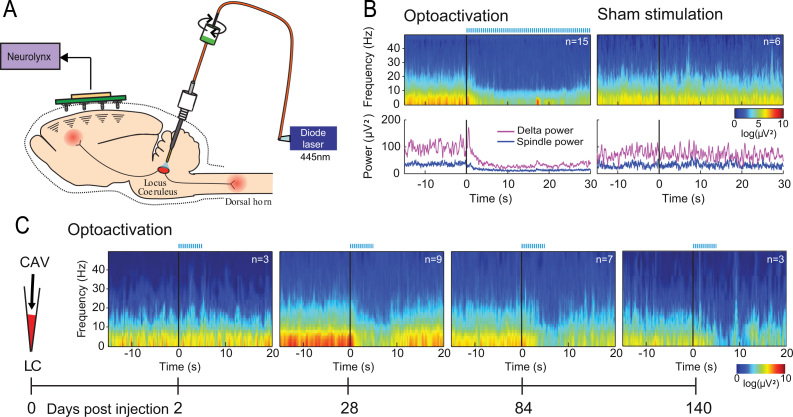
Long-term transduction of Locus Coeruleus with ChR2 allows repeated awakenings from sleep. (A) Schematic of EEG recording from a transduced animal showing implanted optic fiber and EEG/LFP recording from cortex. (B) Opto-activation of the LC (5 Hz, 100 ms pulses for 30 s) reliably produced time locked arousal from sleep showing a loss of EEG spindles (band 10–16 Hz) and also loss of delta power (band 0.4–4 Hz). In contrast sham stimulation (without light) produced no effect. (C) Arousal from sleep was first seen at 4 weeks post transduction and continued to be evoked with the same stimulus parameters (5 Hz, 5 s) until 140 days (post-hoc histology shown in Fig. 1B).

The demonstration of reliable opto-activation of LC neurons *in vivo* raised the question of whether this activation could produce changes in behavior that were stable over time. We used the ability of LC activation to promote sleep-wake transitions as an assay (Carter et al., 2010). Unilateral LC activation reliably produced brief sleep-wake transitions in response to short periods of stimulation (Fig. 2, 5 Hz train for 5 s). Electroencephalogram monitoring showed that LC stimulation produced a loss of delta power and cessation of spindle activity. The ability to produce arousal from sleep was maintained for >6 months indicating that the functional expression of ChR2 was stable (Fig. 2C, *n*=3 rats). Robust, maintained ChR2-mCherry expression was confirmed on post hoc histological examination (shown in Fig. 1A).

### 2.5 Retrograde transduction of brainstem NA neurons after LC injection

**Fig. 3:**
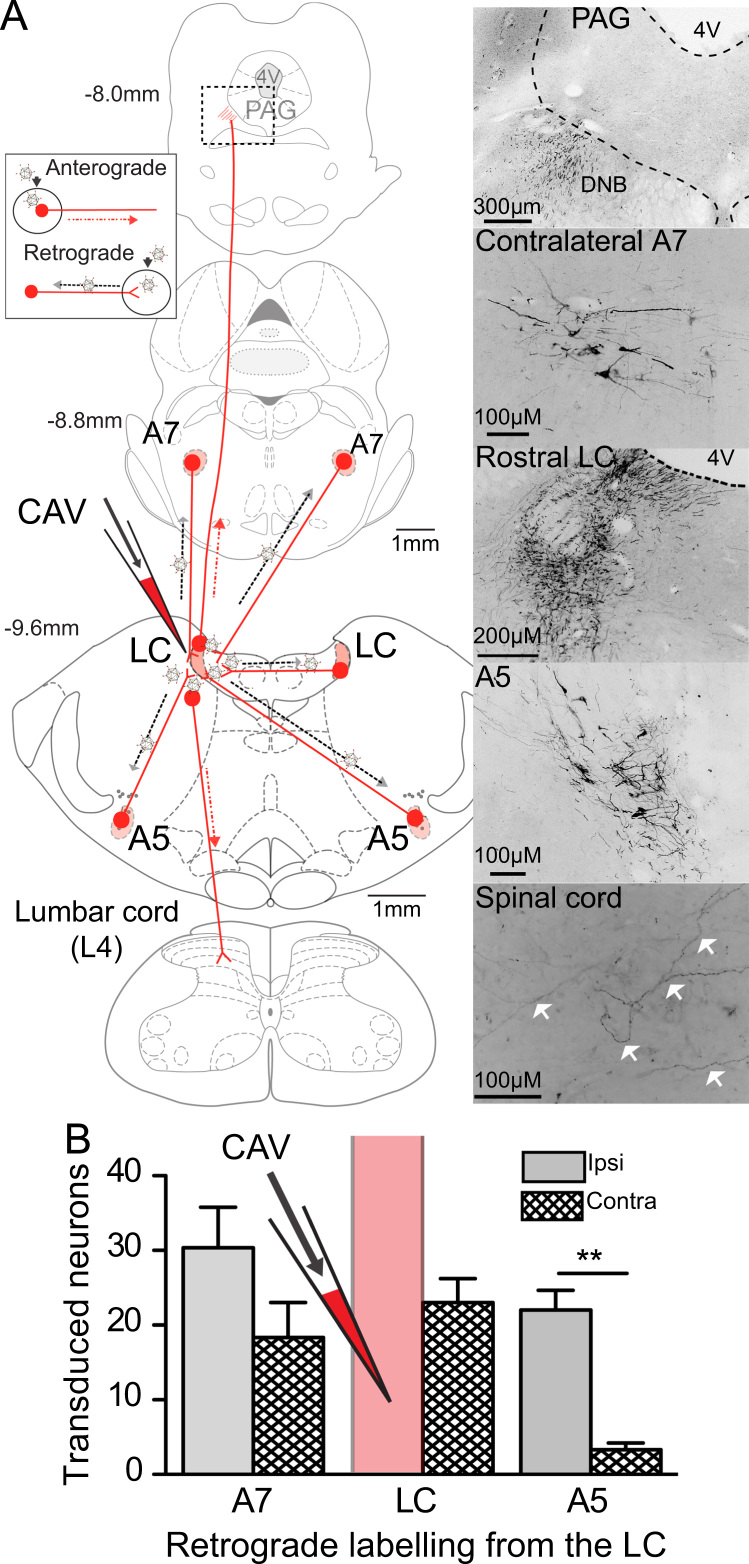
CAV2-PRS-ChR2-mCherry retrogradely transduces NAergic neurons within the pons. (A) Direct injection of CAV2-PRS-mCherry-ChR2 to the LC transduced NAergic neurons locally and also retrogradely transduced neurons in the contralateral LC and in the A7 and A5 cell groups both ipsilaterally and contralaterally. Additionally axon fibers were anterogradely filled with mCherry seen ascending through the pons from the LC through to the midbrain (in the Dorsal noradrenergic bundle (DNB) and also descended to reach the spinal cord. (B) Shows numbers of retrogradely transduced neurons across the noradrenergic cell groups (*n*=3 rats, mean ±SEM, cell counts Abercrombie corrected). All photomicrographs show native mCherry fluorescence converted to inverted grayscale for clarity.

Unilateral LC injection of CAV2-PRS-ChR2-mCherry (*n*=3) induced mCherry expression at distant sites in the pons (Fig. 3). This transduction was restricted to DBH+ neurons, which were found in the contralateral LC and bilaterally in A5 and A7 cell groups (Fig. 3A). This expression is consistent with retrograde transduction by CAV2 given that it occurred over distances of several millimeters (labeling was also noted more distally in the medullary A1/C1 and A2 cell groups). CAV2 transduction also produced strong anterograde axonal labeling with dense bundles of mCherry containing fibers running ipsilaterally from the LC (Fig. 3A) to pass rostrally through the midbrain in the dorsal NAergic bundle; axons were also seen extending to the cerebellum and caudally to both sides of the spinal cord.

### 2.6 Transduction of pontospinal NAergic neurons

**Fig. 4:**
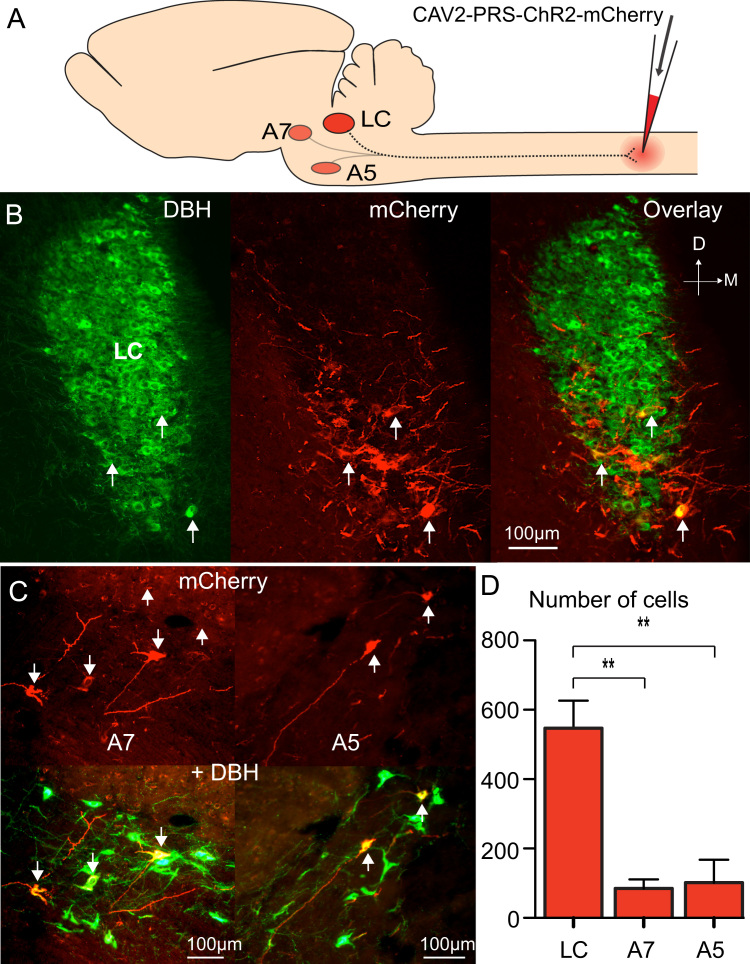
CAV2 efficiently targets the pontospinal NAergic neurons. (A) Spinal microinjection of CAV2-PRS-ChR2-mCherry to the dorsal horn at L4-5 level labeled a subset of NAergic neurons in the LC (B) as well as in the A5 and A7 groups (C). The majority (74%) of the pontospinal neurons (D) were in the LC with the remainder in the A5 and A7 cell groups (*n*=3 rats, one way ANOVA with Bonferroni post hoc tests).

Given that retrograde transduction was found within the brainstem, the next step was to test whether the vector could transduce pontine NAergic neurons over longer distances from the spinal cord. Two weeks after lumbar spinal injection of CAV2-PRS-ChR2-mCherry (titre 0.9×10^10^ TU/ml), retrograde labeling of NAergic neurons was seen in the pons (479±161 neurons, n=3 rats, Fig. 4). The majority of pontospinal neurons were in the ventral LC (75%) with the remainder in A5 and A7 cell groups (Table 2). Equivalent injections containing ~100-fold more CAV2-PRS-ChR2-mCherry (1.2×10^12^ TU/ml) produced ~50% increase in the number of retrogradely labeled NAergic neurons in the pons (733±170, *n*=3) with a similar distribution across the cell groups (Fig. 4D, Table 2). No labeling was seen in brainstem when the same quantity of CAV2 vector was injected intrathecally at the lumbar level (*n*=3) indicating that transduction requires intra-parenchymal injection and reflects retrograde transport. Examination of the spinal cord in the region of the intraparenchymal injection sites showed no local labeling of neuronal somata (consistent with the specificity of the PRSx8 promoter) and we found little evidence of local scarring, unlike our experience with the highest titres of a hAdV-C5 vector (Howorth et al., 2009a).

**Fig. 5:**
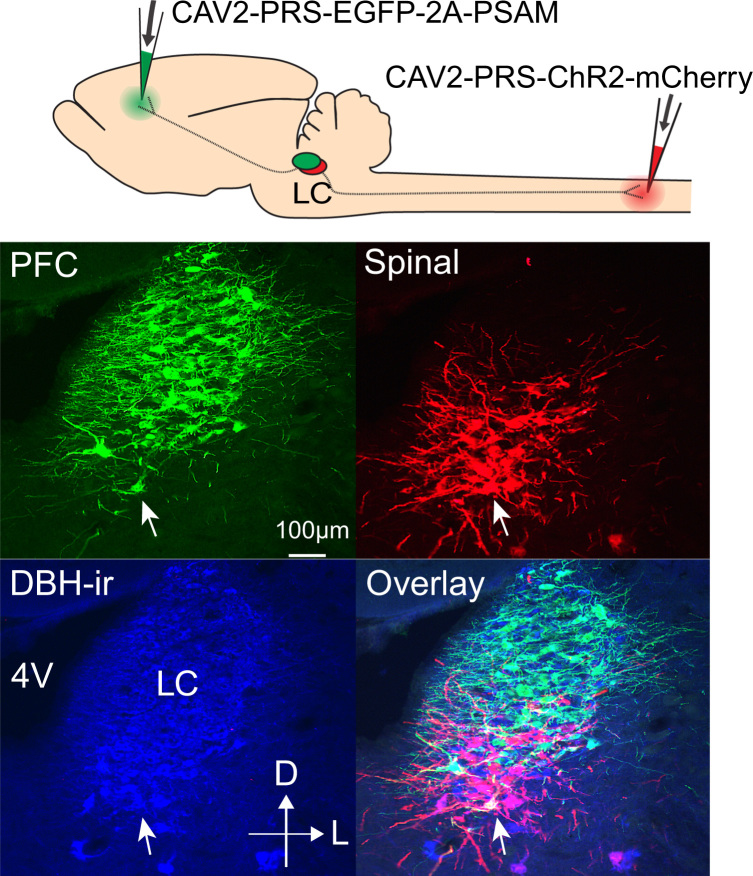
The ps:LC neurons form a distinct module. Paired injections of CAV2-PRS-mCherry-ChR2 and CAV2-PRS- EGFP-2A-PSAM to the lumbar spinal dorsal horn and prefrontal cortex (CG1), respectively, labeled distinct subsets of LC neurons (*n*=3 rats) with the spinal group (red) located more ventrally in the LC with only minimal overlap with those neurons labeled from frontal cortex (green, arrow marks a single double labeled cell body). DBH-ir – revealed with AMCA secondary antibody (blue).

**Fig. 6:**
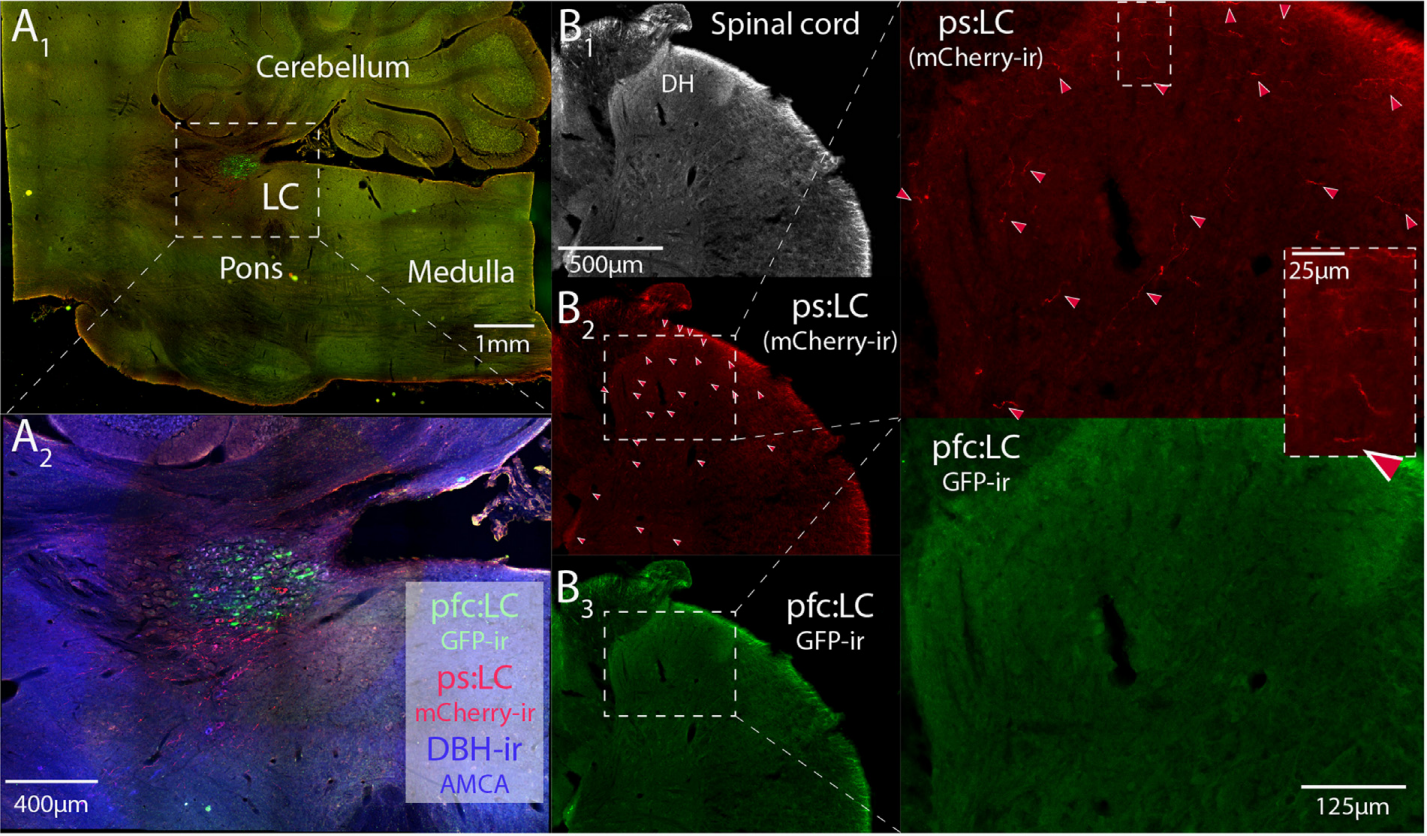
The LC innervation of the spinal cord originates from ps:LC module rather than pfc:LC. (A) Paired injections of CAV2-PRS-mCherry-ChR2 and CAV2-PRS- EGFP-2A-PSAM to the lumbar spinal dorsal horn and prefrontal cortex (CG1), respectively, labeled distinct subsets of LC neurons shown in parasagittal section – note larger numbers of somata labeled from pfc. (B) Examination of spinal cord sections (L2 dorsal horn shown) demonstrated the presence of numerous mCherry-ir axons (B_2_) originating from transduced ps:LC neurons with a complete absence of EGFP-ir fibres (B_3_) indicating that the pfc:LC neurons do not contribute to the spinal innervation. Note the increased density of axons seen in the superficial dorsal horn. Red arrows indicate mCherry-filled axons.

**Fig. 7:**
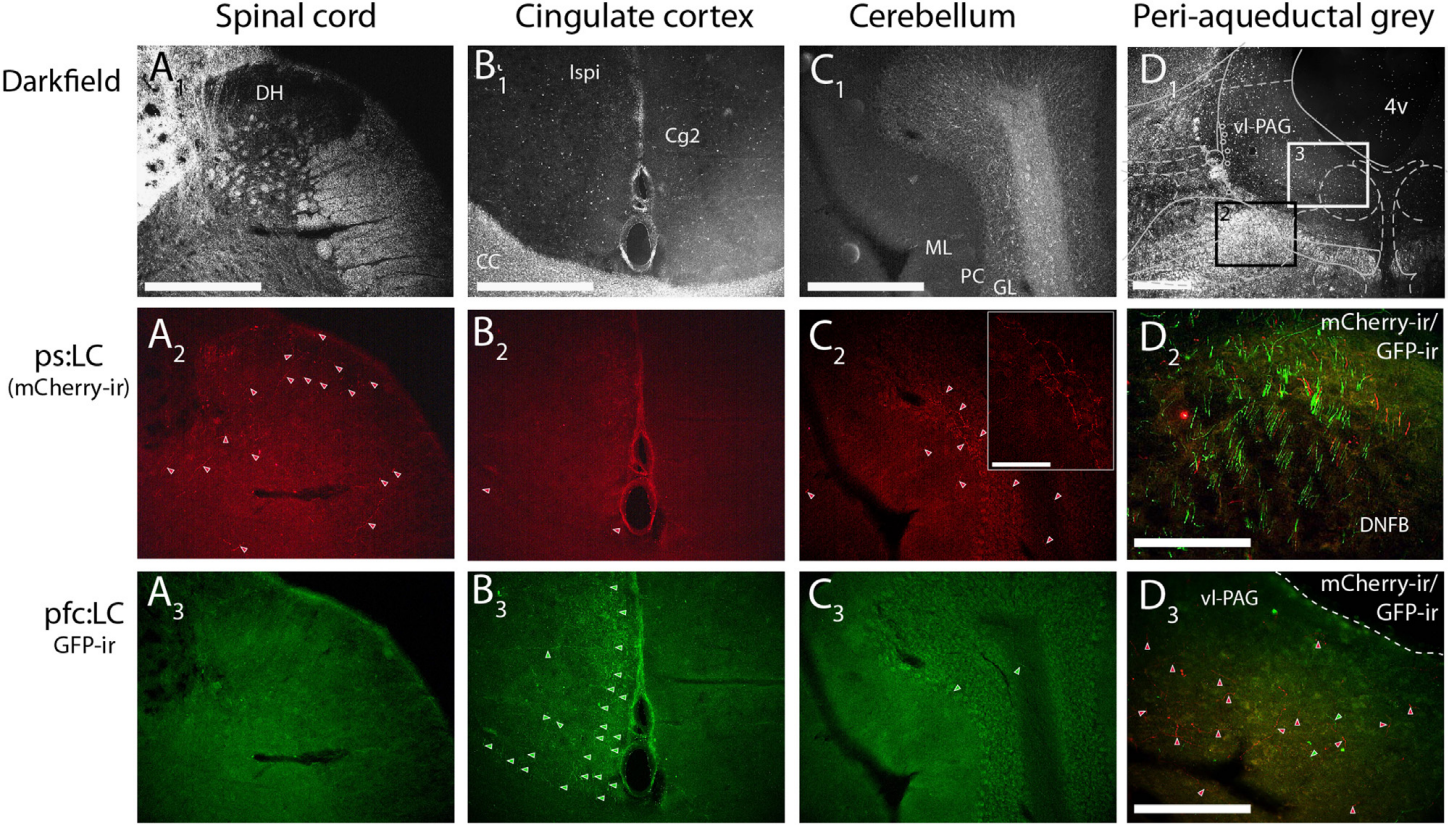
Selective projection targets of ps:LC and pfc:LC neurons. Rats (*n*=3) received dual injections to the lumbar spinal cord (bilateral to dorsal horn, CAV-PRS-ChR2-mCherry) and prefrontal cortex (unilateral to CG1, CAV-PRS-EGFP-2A-PSAM) allowing the comparison of the projection targets of the filled axons (after immunohistochemistry). This showed as expected that the spinal cord (A) and prefrontal cortex (B) are selectively innervated by axons originating from ps:LC or pfc:LC neurons respectively with only sporadic axons from the other module. The cerebellum (C) was predominantly innervated by the ps:LC neurons again with only sporadic fibers from the pfc:LC neurons. Examination of midbrain sections (D) showed that the dorsal noradrenergic bundle contained axons from both the pfc:LC and the ps:LC modules with some co-localization (D_2_, shown as overlaid GFP and mCherry). However, in the nearby PAG (D_3_) the large majority of the axons were from the ps:LC module indicting a specificity of innervation. Green and red arrows indicating GFP- or mCherry-filled axons. All scalebars=500 µm (except D2, D3=250 µm). DH – Dorsal horn, CG2 Cingulate cortex, ML – molecular layer; PC purkinje cell layer; GL – granule cell layer; vl-PAG – ventrolateral PAG, 4V – 4th Ventricle.

Dual injections with CAV2-PRS-ChR2-mCherry and CAV2-PRS-EGFP-2A-PSAM into the dorsal horn of the lumbar spinal cord and prefrontal cortex (CG1) demonstrated a spatial segregation of the retrogradely labeled neurons into ventral (spinal) and dorsal (PFC) groups evident in coronal (Fig. 5) and para-sagittal sections (Fig. 6). Only a small proportion of the ps:LC neurons were double labeled from the PFC (5.8±1.9% double labeling, *n*=3 rats, Fig. 5). This approach also revealed contrasting distributions of the anterogradely filled axonal projections seen from each cell group (Fig. 6, Fig. 7 and Table 3). The ps:LC neurons showed the expected projections to the spinal cord (unlike the pfc:LC, Fig. 6) but also projected to the medullary raphe, periaqueductal grey, cerebellum, inferior olive and anteroventral nucleus of the thalamus, all regions which received little or no input from the pfc:LC (Fig. 7). In contrast areas like the hippocampus showed little innervation from the ps:LC while labeling was clearly visible from the pfc:LC. A small number of double-labeled axonal fibers were seen in the ascending noradrenergic bundle with a few sparsely distributed in the cortex and the spinal cord consistent with the 5% of neurons showing somatic double labeling (Fig. 7). These data indicate successful targeting of a demarcated subgroup of LC neurons with a distinct sets of axonal projection targets.

### 2.7 Optogenetic activation of pontospinal LC neurons

**Fig. 8:**
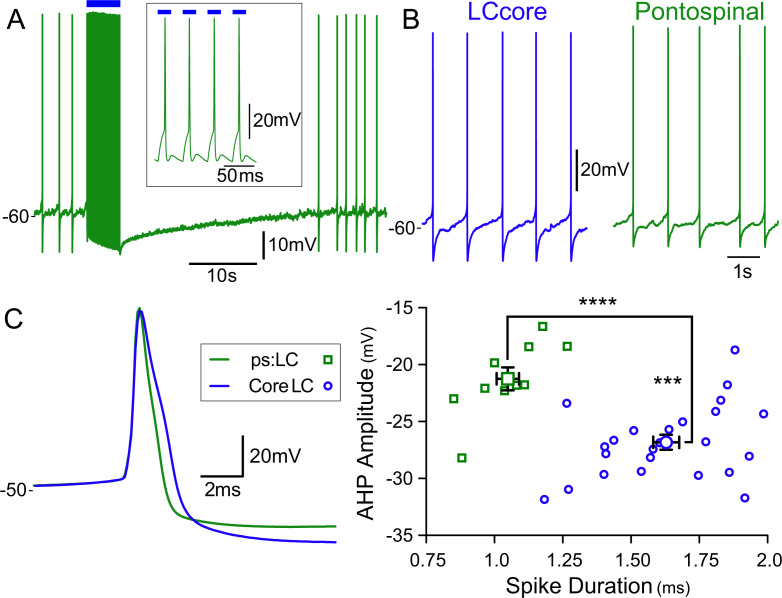
ps:LC neurons have distinctive electrophysiological characteristics. (A) Whole cell recordings from retrogradely transduced ps:LC neurons showed they could be opto-activated (20 ms×10 mW, 25 Hz, expanded inset). (B) Recordings from neurons in ps:LC and in the core of the LC; both showing healthy patterns of activity. (C) Action potential morphologies from two representative neurons (green – ps:LC, blue – LC core) and scatter plot of action potential duration plotted against AHP amplitude for each LC neuron showing that ps:LC neurons (green, n=9) had significantly shorter spike durations and smaller AHPs then neurons in the core of the LC (blue, *n*=24). (**** - *P*<0.0001; *** - *P*<0.001, unpaired Student׳s *t*-test).

Recordings of spinally transduced LC neurons in pontine slices showed light-evoked ChR2 inward currents (147±72 pA steady state current at *V*_h_ −60 mV, *n*=8, p28–35) that allowed action potential generation in current clamp recordings (Fig. 8). The delay to first spike after the onset of illumination and the jitter around that value showed a clear relationship to the magnitude of the ChR2 current. Strongly transduced neurons fired reliably within several milliseconds of pulse onset whereas neurons with lower levels of transduction required longer light pulses (often >50 ms) and showed more variation in the latency (Supplemental Fig. 1). A similar phenomenon was also noted in the directly transduced neurons. Nonetheless the level of ChR2 transduction in the ps:LC neurons was still sufficient to produce a substantial 8.32±2.4 fold increase in action potential discharge with longer light pulses (>100 ms). These observations led us to use longer pulses of illumination to opto-identify ps:LC neurons *in vivo* (Section 2.8).

On electrophysiological grounds, these transduced neurons appeared healthy *in vitro* with normal spontaneous firing activity. In comparison to LC neurons transduced after direct injection (located in the core of the nucleus) they showed many similarities (see Table 1) but with markedly shorter action potentials (1.05±0.04 *vs* 1.63±0.05 ms, *P*<0.0001) and smaller AHPs (−21.3±1.0 *vs* −26.8±10.7 mV, *P*<0.001, Fig. 8C). This indicates an electrophysiological specialization of spinally projecting LC neurons, like that reported for subgroups of cortically projecting LC neurons (Chandler et al., 2014).

### 2.8 Opto-activation of the ps:LC module *in vivo*

**Fig. 9:**
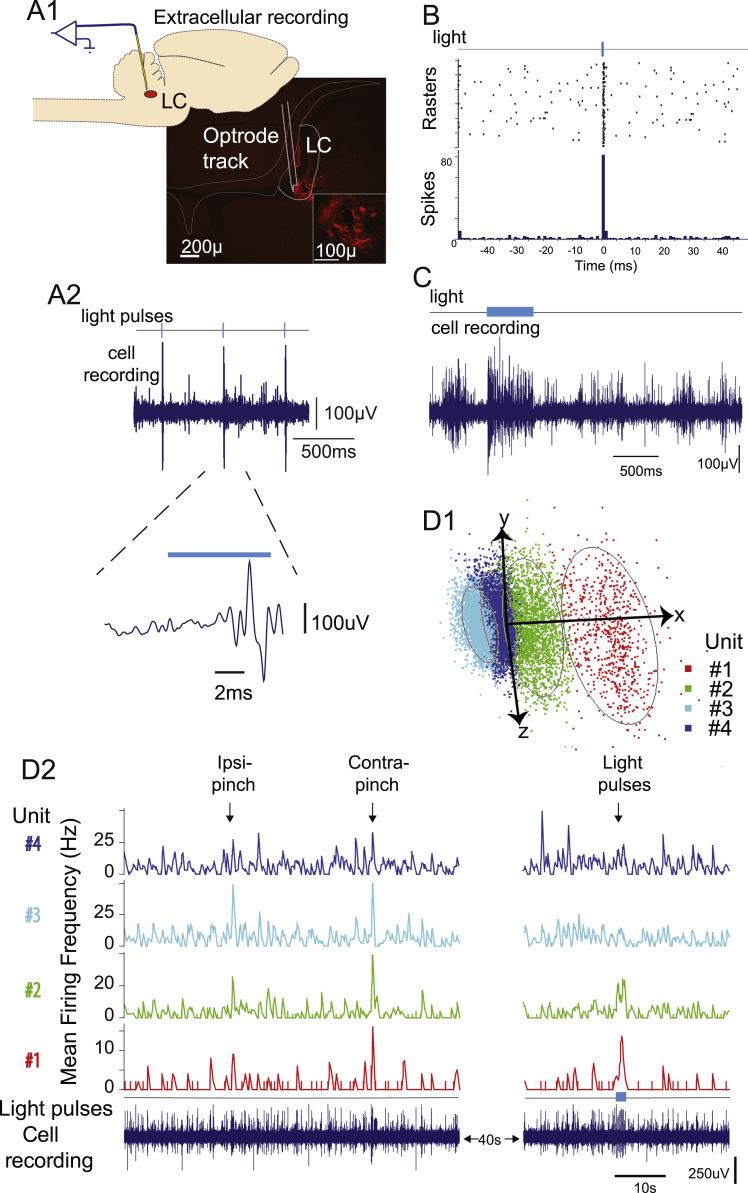
*In vivo* recording from ps:LC neurons. (A) Spinally transduced LC neurons were identifiable *in vivo* on the basis of their response to light pulses (recording position in LC shown post hoc in A_1_) (B) ps:LC neurons could be entrained to discharge action potentials by light flashes (445 nm, 2 Hz, 7 ms pulses, 7 mW) and could also be driven to fire at a sustained higher rate by more prolonged illumination (C, 500 ms). (D) In this same recording four LC units were individually discriminable by wavemark templating and shown here separated by principle component analysis (D_1_, ovoids mark 2SD from mean). The four units shown D2 all showed excitatory-inhibitory responses to contralateral hindpaw pinch but only two were identified as being spinally projecting by their excitatory response to light pulses (445 nm, 5 Hz, 11 mw, 20 ms).

*In vivo* extracellular recordings from the LC in spinally injected rats (*n*=6, 3–4 weeks after transduction) allowed the identification of ps:LC neurons by opto-activation (see Fig. 9). The majority of the recorded ps:LC neurons (*n*=6, located 6.0±0.2 mm deep to the pial surface) were spontaneously active *in vivo* 1.4±0.6 Hz (*n*=5/6). The firing rate of ps:LC neurons was significantly slower than that seen in directly transduced LC neurons (3.7±0.8 Hz, *p*<0.05) that were located more dorsally in the LC (5.3±0.12 *vs* 6.0±0.2 mm, *P*<0.05). The firing frequency of the ps:LC neurons was increased 5–6 fold by a single light pulse (8.0±2.8 Hz, *P*<0.05, *n*=6, 20 mW×1 s). Of the cells tested, over half showed a 1:1 entrainment by short pulses (*n*=4, Fig. 9B) and the remainder required longer pulse durations (>100 ms) to increase their firing. The ps:LC neurons were all excited by noxious stimuli *e*.*g*. pinch applied to the contralateral hindpaw (Fig. 9D). Therefore the ps:LC neurons can be driven to fire *in vivo* by optical stimulation and they have distinctive patterns of ongoing activity.

## 3 Discussion

Through the use of retrograde optogenetics, we show that the spinally projecting LC neurons form a topographically and functionally distinct subset of the nucleus. These are distinguishable from a pool of LC neurons projecting to the pre-frontal cortex on the basis of their location and distinctive pattern of output projections. As such, this indicates a modular output organization of the LC neurons with a particular functional specialization of the NAergic neurons involved in the regulation of nociception.

In undertaking this study we faced an issue common to the study of all long-range neuromodulator systems that require the influence of a specific projection circuit to be independently manipulated. This specificity of functional control can be achieved through the use of viral vectors capable of retrograde transport (Hnasko et al., 2006, Howorth et al., 2009a, Howorth et al., 2009b). The introduction of optogenetics has widened the scope for such functional manipulation, however, reliable retrograde optogenetic control is still challenging as the small conductance of the ChR2 pore requires a high level of protein expression (Boyden et al., 2005). In the current study we employed CAV2 vectors for retrograde transduction of LC neurons (over distances of >100 mm) which produced sufficient expression of ChR2 to allow opto-activation. Retrogradely transduced LC neurons had smaller light-evoked ChR2 currents (~30%) than directly transduced cells, but this was still sufficient to allow spikes to be reliably evoked. The transduction of LC neurons by CAV2 appeared to have no effect on cellular health based on electrophysiology and on the ability to evoke sleep-wake transitions for periods >6 months.

CAV2 vectors transduced 3–4 fold more pontospinal NAergic neurons than an equivalent hAdV-C5 with the same PRSx8 promoter element and fluorophore (Howorth et al., 2009a). Given that CAV2 and hAdV-C5 gain access to neurons by a common cellular pathway (a coxsackievirus adenovirus receptor); this increased efficacy is likely due to the selectivity of CAR use on neurons by CAV (Salinas et al., 2009), and other capsid characteristics such as the global charge and flexibility of the fibers (Bru et al., 2010). The pattern of pontospinal NAergic transduction seen with CAV2 across the three cell groups LC, A5 and A7 was similar to that previously seen with hAdV-C5 (Howorth et al., 2009a). However, these data revise upwards the estimate of the proportion of LC neurons with spinal projection to ~15% (based on 3,268 LC neurons (Loughlin et al., 1986)) a similar number to that seen with fluorogold labeling from the spinal cord (Howorth et al., 2009a). The spinal injection of CAV2 also transduced ~25% of the A7 neurons and ~5% of A5 cells (based on total counts from (Howorth et al., 2009a)). The low proportion of A5 neurons likely relates to the lumbar injection site below their major projection targets in the sympathetic cell column (Bruinstroop et al., 2012).

Although LC neurons possess long, extensively ramifying axons there is some evidence of organizational specificity with groups of LC neurons projecting to specific targets that process particular sensory signals (Berridge and Waterhouse, 2003). An exemplar of this principle is that the LC directly suppresses the spinal transmission of nociceptive information by a descending projection to the spinal dorsal horn (Howorth et al., 2009a, Jones, 1991, Millan, 2002, Pertovaara, 2006, Yoshimura and Furue, 2006) but these neurons also project to the thalamus and to cortical territories indicating the potential for multilevel modulation of sensory input. Using the CAV2 vector for retrograde transduction, we found a similar pattern of targeting by fibers from the ps:LC neurons to supraspinal structures. However, and notably, ps:LC axonal terminals were found in several regions that were non-overlapping with the distribution of fibers belonging to LC neurons labeled from the PFC. Specifically fibers in the medullary raphe, PAG, cerebellum and inferior olive originated almost exclusively from ps:LC neurons whereas the hippocampus had no fibers from the ps:LC neurons but an innervation was seen from the pfc:LC neurons. In turn the spinal cord had almost no innervation from pfc:LC neurons; although the pfc does get a weak innervation from the ps:LC (hence the 5% of double labeled LC neurons). This distinctive distribution of axons argues in favor of a degree of anatomical specificity of the output projections from the LC.

It is also apparent from our results that the pfc:LC neurons also supply collaterals to many other cortical and subcortical territories in agreement with a recent set of elegant studies of LC input-output organization (Schwarz et al., 2015). Intriguingly, the medullary projecting LC neurons reported in that study had a distinct set of synaptic inputs that were different from the rest of the LC suggesting that this may be a functionally distinct population. We posit that these LC neurons retrogradely labeled from the medulla are likely to be drawn from the distinctive population of ps:LC neurons given that they also have a ventral location in the nucleus. Therefore a parsimonious interpretation of our apparently contrasting findings is that there are at least two subsets of LC neurons, one group projecting to the spinal cord and selected brainstem and supratentorial structures and a second larger component innervating much of the forebrain (the degree of sub-division within this population is currently an area of active debate (Chandler et al., 2014)).

The ps:LC neurons had distinctive electrophysiology with shorter action potentials and smaller afterhyperpolarizations. Although in all other respects their properties were similar to transduced neurons recorded in the core of the LC, the differences in their action potential morphology are likely to increase their ability to transduce high frequency synaptic drives – as seen with noxious inputs to the LC. This specificity of intrinsic properties has parallels with the recent studies by Chandler and colleagues (2014) who have found electrophysiological and synaptic distinctions between LC neurons projecting to different cortical territories.

We identified ps:LC neurons *in vivo* on the basis of the ability to increase their firing rate on illumination with blue light (445 nm). We used longer duration light pulses (1 second) to effectively excite the LC neurons a similar protocol to our previous *in vivo* optogenetic study of LC neurons directly transduced with a lenti-viral vector (Hickey et al., 2014). This light pulse duration is longer than that previously used to identify neurons in the forebrain *in vivo* (Lima et al., 2009) and only around half of our identified neurons showed reliable, phase-locked discharge to short pulses (<50 ms). We had previously noted from slice recordings that the latency to spike discharge and the jitter about this value were strongly dependent upon the magnitude of the ChR2 current in individual neurons and although all neurons showed a robust increase in firing in many this could not be generated by pulses of duration <50 ms. This characteristic likely also reflects in part the slow membrane time constant and presence of strong rectifying conductances in LC neurons. The use of long pulses and the lack of precise phase locking of spike discharge raises the question of whether these excitations could be indirectly mediated. However, given the selectivity of transduction of NA neurons seen with CAV-PRS-ChR2-mCherry then this would have to be mediated by an excitatory adrenoceptor within the LC. In all of our *in vitro* recordings from non-transduced LC neurons we never found evidence of such effects when optoexciting transduced neighbours (*n*=82 non-transduced LC neurons) but frequently observed inhibitions (paper in preparation). On this basis we feel justified in describing these neurons as being opto-identified *in vivo*.

The ps:LC neurons in adult rats *in vivo* had slower ongoing firing rates than LC neurons identified in the more dorsal core of the nucleus (1.4 *vs* 3.7 Hz; in close agreement with Guyenet (1980) who found 1.2 *vs* 2.6 Hz for coerulospinal versus coerulocortical neurons) consistent with the notion that they have distinct afferent drives (as we found no difference in their spontaneous discharge *in vitro)*. The ps:LC neurons were noci-responsive as previously suggested on the basis of c-fos expression (Howorth et al., 2009a) and had been reported electrophysiologically (Guyenet, 1980) and in line with that reported for LC neurons as a whole (Cedarbaum and Aghajanian, 1978b, Guyenet, 1980). Such noci-responsiveness is a requirement for these neurons to play a role in regulating responses to noxious stimuli. Taken together with the *in vitro* findings the distinctions between subsets of LC neurons further challenge the notion of the LC as a homogenous cluster of neurons and instead indicates functional specialization.

The findings of ps:LC modularity may also help account for the recent observation that after direct transduction of the LC with ChR2 there was bidirectional modulation of nociception with an analgesic effect evoked from a ventral portion of the nucleus (Hickey et al., 2014)-the site of the pontospinal somata. Whereas pro-nociceptive effects of LC stimulation were evoked from the more dorsal part of the nucleus where the forebrain projection arises (Swanson, 1976) – which may be acting to promote attention to the stimulus (Berridge and Waterhouse, 2003). Therefore targeting the pontospinal LC projection to produce analgesia may minimize the side-effects associated with conventional systemic pharmacological intervention (Hughes et al., 2015)

This study identifies CAV2 vectors as useful tools for retrograde optogenetics facilitating functional deconstruction of long-range neuromodulator circuits. Our data support the principle that the locus coeruleus is functionally organized into modules; with the pontospinal module having distinctive properties – perhaps reflecting a different developmental origin as suggested for some ventral LC neurons in mice (Robertson et al., 2013). The application of such retrograde optogenetic approaches may enable the functional discrimination of the roles of the LC modules to be determined in behaving animals as well as at a cellular level.

## 4 Experimental procedures

All procedures conformed to the UK Animals (Scientific Procedures) Act 1986 and were approved by the University of Bristol local Ethical Review Panel. Experiments were performed on male Wistar rats. Animals were housed, with an enriched environment, under a standard 12 h light/dark cycle, with *ad libitum* access to food and water.

### 4.1 CAV2 vector construction

A transgene cassette containing PRS-ChR2(H134R)-mCherry-WPRE was excised from p-Le-PRS-ChR2(H134R)-mCherry (gift from Dr Ruth Stornetta, University of Virginia) with *Pac*I/*Kpn*I double digestion and was blunt-ended with T4 DNA polymerase (New England Biolabs). This transgene cassette was then ligated into a pre-cut (*Eco*RV/*Kpn*I) and blunt-ended CAV2 shuttle vector pTCAV2-12a to generate pTCAV2-PRS-ChR2-mCherry. The internal *Not*I site between ChR2 and mCherry was removed by site-directed mutagenesis (Quickchange, Agilent Technologies). The transgene unit PRS-ChR2(H134R)-mCherry-WPRE was then transferred into the CAV2 genome through homologous recombination between the shuttle vector pTCAV2-PRS-ChR2-mCherry and the CAV2 genomic construct pTG5412 in BJ5183 cells (Agilent Technologies) following the manufacturer׳s protocol. A second CAV2 vector (CAV-2-PRS-EGFP-2A-PSAM) was designed and used for the double vector injection experiments. This vector contained a cassette for the expression of EGFP with a 2A linker peptide and the engineered chemogenetic receptor PSAM_L141F,Y115F-5HT3HC_ (Magnus et al., 2011). A plasmid containing CMV-EGFP-2A-PSAM was custom synthesized by GeneArt AG. The expression cassette EGFP-2A-PSAM was excised by AgeI/HpaI digest and ligated into pTCAV-PRS-ChR2-mCherry that was cut with AgeI/EcoRV to remove ChR2-mCherry. The resulting pTCAV-12a-PRS-EGFP-2A-PSAM was purified, then digested with BamHI/NotI for homologous recombination of PRS-EGFP-2A-PSAM into the SwaI linearized CAV2 genome (pCAVΔE3Sce) as above. CAV2 vectors expressing mCherry or EGFP under the control of the CMV promoter were used in control experiments. CAV2 vector generation and amplification employed previously described methods (Ibanes and Kremer, 2013).

#### 4.1.1 Vector titration

Vector stock titre was determined by an immuno-assay of functional transduction similar to that detailed previously for hAdVs (Howorth et al., 2009a). Briefly, serial dilutions of vector were used to transduce DKZeo cells seeded in a 12-well-plate 24 h earlier (1 ml/well). Two days post-inoculation cells were fixed with methanol at −20 °C for 20 min. After PBS washes the cells were probed with a mouse anti-CAV2 primary antibody (Investcare-Vet; 1: 1000 in PBS with 0.3% BSA for 2 h at 37 °C). After PBS washes the cells were incubated with anti-mouse-horseradish peroxidase secondary antibody (1:1000; Abcam) for 1 h at room temperature. The cells were stained with an enhanced DAB Substrate kit (Pierce, Thermo Scientific) according to manufacturer׳s protocol. The number of DAB positive cells/well was counted and titre was calculated as transducing units/ml of viral solution (TU/ml). A second assay of physical particles (pp/ml) was also performed for the vector (Kremer et al., 2000) allowing comparison of titres with previous CAV2 studies.

### 4.2 Stereotaxic injection

Vector injections followed the methods previously described (for LC (Hickey et al., 2014)) and the lumbar dorsal horn (Howorth et al., 2009a, Howorth et al., 2009b). Briefly, rats were anesthetized for recovery surgery with ketamine (5 mg/100 g body weight i.p, Vetalar, Pharmacia, UK) and medetomidine (30 µg/100 g body weight i.p, Domitor, Pfizer, UK).

#### 4.2.1 Direct LC injections

A burr hole (∅ 1.0 mm) was made over the LC (300 g rats) at stereotaxic coordinates from lambda, AP: −2.1 mm, ML: 1.2 mm. A glass micropipette was advanced with a 10° rostral angulation to a depth of 5.5 mm from brain surface. Three vector injections (0.3 µl/each, at a speed of 0.25 µl/min) were made at 5.3, 5.5 and 5.8 mm depths. To transduce LC neurons in weaner rats (p21) prior to *in vitro* brain slice recordings a burr hole was made at AP: −1.0 mm; ML: −1.0 mm (relative to lambda). Four vector injections (0.25 µl each) were made at depths of 4.6, 4.9, 5.2 and 5.5 mm along the track with 10° rostral angulation.

#### 4.2.2 Lumbar spinal injections

The spinous processes of T13 and L1 were located and a laminectomy was performed through a midline skin incision to access spinal segments L3–L4. The vertebrae were clamped in a spinal clamp (Narishige, Japan) to ensure stability. For each injection site, 0.4 µl CAV2 vector was injected into the dorsal horn 400 µm lateral to the midline and 400 µm deep from the dorsal surface at a rate of 0.25 µl/min, using a calibrated micro-capillary pipette with a tip diameter of ~20 µm. Two pairs of bilateral injections, 500 µm apart rostrocaudally, were made into the L3–4 spinal segments. For experiments where recordings from pontine slices were planned, injections were made at age P21 into L3–L4, 250 µm lateral to the midline and 250 µm deep.

#### 4.2.3 Prefrontal cortical injections

Direct injections of the vector into Cg1 were performed through a limited craniotomy over prefrontal cortex. A series of eight injections were made (150 nl/injection, 4×10^11^ TU/ml) down four tracks (two injections per track at 1 and 1.5 mm deep to cortical surface) each 0.6 mm lateral to midline and at 0.8 mm intervals from +1.8 to −0.6 mm relative to bregma.

### 4.3 Guide cannula/ferrule implant

To allow insertion of an optical fiber-ferrule for opto-activation during behavioral experiments *in vivo* a 22G stainless steel guide cannula (C313G, Plastics-one, Roanoak, USA) was implanted unilaterally through a burr hole to sit above the LC (4.0 mm deep to brain surface, angled with the tip facing 10° rostral) and was secured to the skull surface with dental cement+skull screw (Zhang et al., 2010). The guide cannula was closed with a dummy cap until the time of the experiment. Alternatively for subsequent *in vivo* cell recordings under anaesthesia a skull screw (0–80×1/16, Plastics-one, Roanoak, USA) was inserted into the burr hole and removed leaving an access route for cell recordings (2–3 weeks post-transduction).

### 4.4 Sleep-wake studies: pre-frontal cortex local field potential recordings

These studies used Long-Evans rats (400 g, males, *n*=3) as these are the strain currently employed in related sleep-wake studies in our groups. The rats were anaesthetized with isoflurane and had direct stereotaxic injections of CAV2 to their left LC (as detailed above). They were then implanted with either screws (M1.2×3 mm) above the frontal cortex (with reference and ground over cerebellum), connected to an EEG electrode interface board (EIB 18, Neuralynx, MT; see Fig. 2) or a custom built tetrode-microdrive targeting the medial prefrontal cortex (+3.2 mm from bregma, 0.6 mm lateral, 1.5–3.0 mm ventral to brain surface; see Fig. 2 and methods after (Gardner et al., 2013)). The assembly also contained an independent optical fiber ferrule lowered *via* a guide cannula to the LC. Tetrodes were fabricated from twisted bundles of 13 μm polyimide-insulated nichrome wire (Kanthal, Sweden).

Electrophysiological data were acquired using Digital Lynx hardware and Cheetah software (Neuralynx) while rats were at rest or asleep in their home cage inside a dimly lit, sound-attenuating chamber. Local field potential recordings were made from a single tetrode wire in the mPFC referenced to a silent wire in the motor cortex and sampled at 2 kHz and band-pass filtered at 0.1–600 Hz. Behavior was continuously monitored *via* four video cameras. The optical fiber ferrule (Thorlabs) was connected *via* a rotary joint (Doric) for opto-activation of the LC (445 nm diode laser, Omicron Phoxx, 20 mW, 50 ms, 1–8 Hz). The fiber was lowered towards the LC from 5 mm deep and stimuli were applied until it was possible to reliably evoke sleep wake transitions with short periods (≤30 s) of opto-activation (after (Carter et al., 2010)). All analyses were performed in MATLAB (MathWorks, MA). Multitapered spectral analyses (Mitra and Pesaran, 1999) were performed using the Chronux toolbox (www.chronux.org). Delta and spindle power were measured using the absolute value of the Hilbert transform over the ranges of 0.4– 4 Hz and 10–16 Hz respectively.

### 4.5 Pontine slice preparation

Brainstem slices were prepared as previously described (Hickey et al., 2014) from rats 7–14 days after vector injections (aged 28–35 days post-natal). In brief, rats were terminally anesthetized with halothane 5% before decapitation. The brainstem was removed and bathed in ice-cold dissection artificial cerebrospinal fluid (aCSF) (composition identical to the recording aCSF except NaCl was reduced to 85 mM and substituted with 58.4 mM sucrose). The brainstem was blocked, glued to the vibratome stage (Dosaka LinearslicePro, DSK, Japan) ventral surface down and 300 µm thick horizontal slices were cut. These were transferred to a holding chamber at room temperature and allowed to recover for a minimum of 1 h in carbogen bubbled aCSF (in mM: NaCl (126), KCl (2.5), NaHCO_3_ (26), NaH_2_PO_4_ (1.25), MgCl_2_ (2), CaCl_2_ (2) and D-glucose (10), pH 7.3, osmolality 290 mOsm/L).

### 4.6 Patch clamp electrophysiology

For recordings slices were transferred into the chamber of an upright fluorescence microscope (DMLFSA, Leica Microsystems, Heidelburg, Germany) and superfused with aCSF (2–3 ml/min) at a temperature of 35 °C. Patch pipettes (resistances of 4–7 MΩ) pulled from borosilicate glass (GC120, Harvard Apparatus) were filled with an internal solution (in mM: K Gluconate (130), KCl (10), Na-HEPES (10), MgATP (4), EGTA 0.2 and Na_2_GTP (0.3)). Transduced neurons were identified under epifluorescence illumination (Chroma filter set 41034) by the presence of membrane-bound mCherry fluorescence. Neurons were targeted for whole cell recordings under gradient contrast illumination (Dodt and Kuba, 1990) and the pipette was maneuvered onto the cell surface using a 3D-manipulator (SM5, Luigs and Neumann, Germany). Recordings from LC neurons were made in current and voltage clamp modes (Axon Multiclamp 700A, Molecular Probes, USA). All membrane potentials were corrected for a junction potential of 13 mV. Signals were low pass filtered (3 kHz cut off), digitized at a sampling frequency of 10 kHz (power1401, Cambridge Electronic Design, UK) and stored on PC using Spike2 software (CED).

The threshold for action potential discharge was determined as the point at which the rate of change of membrane potential exceeded 7.5 V/s and all spike parameters were measured with reference to this point (using a custom Spike2 script). Light was pulsed onto the cells (10 mW) using a focally placed optical fiber (∅400 um in diameter, pigtailed to a 473 nm LED source; Doric Lenses, Quebec, Canada) positioned close to the LC.

### 4.7 Optostimulation of LC neurons *in vivo*

The methods for cell recording were similar to those reported previously (Hickey et al., 2014). In brief, Wistar rats which had been LC (*n*=5) or spinally (*n*=6) transduced (3–4 weeks previously) were anaesthetized with isoflurane (1.5–3%) until loss of paw withdrawal reflex. The external jugular vein was cannulated and the animal was switched to intravenous anaesthetic (Alfaxalone, 10 mg/ml, 7.5–15 mg/hr, Vetoquinol, UK) before being placed in a stereotaxic frame. Body temperature was maintained using a homeothermic mat (37 °C) and anaesthetic was titrated to a stable, light plane of anesthesia where a moderate withdrawal reflex could be evoked by pinch of the forepaw. A cell recording optrode was fabricated by attaching a tungsten microelectrode (5 MΩ, parylene-c insulated, A–M systems, WA, USA) parallel to an optical fiber (150 µm core, Thorlabs, UK, after (Abbott et al., 2009)) with its tip 250–500 µm ahead of the fiber end. This optrode was lowered using a hydraulic Microdrive (Narishige, Japan) along a track angled 10° rostral into the LC (5.2–6.5 mm deep to the brain surface). The electrode was referenced against a sintered silver chloride pellet placed under the scalp. The signal was amplified (Axon Multiclamp 700A, Molecular devices, USA), filtered (100 Hz to 2–3 kHz), digitized at 10 kHz (micro1401, Cambridge Electronic Design, UK) and stored on a PC for analysis with Spike2 software. In recordings with multiple units then individual spike waveforms were templated and discriminated in Spike2 and confirmed as being independent using principal component analysis.

Recordings were considered to be from LC neurons if they fitted several criteria: (1) large amplitude action potential waveform; (2) duration of action potential (≥1 ms); and (3) spontaneous firing (Cedarbaum and Aghajanian, 1978a, Hickey et al., 2014, Sugiyama et al., 2012). Following identification of cell with LC characteristics they were illuminated with light pulses (445 nm diode laser, Omicron Phoxx, 0.5–25 mW, 1 ms–10 s, fiber calibrated prior to insertion). The change in LC firing rate was compared to the baseline firing rate before onset of illumination.

### 4.8 Tissue fixation

Rats were culled with an i.p. overdose of pentobarbital (20 mg/100 g body weight, Euthatal, Merial Animal Health, UK) and perfused transcardially with saline (0.9%, 1 ml/g), followed by 4% formaldehyde (Sigma) in 0.1 M phosphate buffer (PB, pH7.4, 1 ml/g). The brain±spinal cord were removed and post-fixed overnight in 4% formaldehyde/0.1 M PB before cryoprotection in 30% sucrose at 4 °C. Coronal tissue sections were cut at 40 µm intervals using a freezing microtome (Leica) and either serially mounted on glass slides or left free floating in PB for fluorescence immunohistochemistry (IHC).

### 4.9 Immunohistochemistry

Tissue sections were washed 3 times in 0.01 M PB and permeabilized in 50% ethanol for 30 min at room temperature. After further washes, sections were incubated with primary antibodies (on a shaking platform, room temp) against dopamine β-hydroxylase (Mouse anti-DBH, 1:10,000 (100 ng/ml); Millipore (Chemicon), MAB308) and/or mCherry (Rabbit anti-mCherry, 1: 2000; catalog # 5993, Biovision, USA) in PB containing 5% horse serum (HS) and 0.3% Triton X-100 for 12–24 hours. Tissue sections were thoroughly washed after removal of primary antibodies and incubated with appropriate secondary antibodies conjugated to fluorophores (Alexa Fluor488 donkey anti-mouse and Alexa Fluor 594 donkey anti-rabbit, both at 1:1000 dilution; Invitrogen and AMCA donkey anti-mouse at 1:100–1:250 dilution, Jackson ImmunoResearch labs, USA) in PB with 2% HS and 0.3% Triton X-100 at room temperature for 3–4 h. Sections were mounted on glass slides and coverslipped with FluorSave (±DAPI; Calbiochem, UK) mounting medium. The specificity of the anti-DBH antibody has previously been validated by our lab (Howorth et al., 2009a). The anti-mCherry antibody has been shown to be specific by Western blot (Biovision manufacturers datasheet) and showed an overlapping distribution with mCherry positive cell bodies and amplified the signal in contiguous distal processes; no staining was seen in control tissues without mCherry. Negative controls were routinely run by omitting the primary antibodies.

### 4.10 Fluorescence microscopy

Images were acquired using a Zeiss Axioskop 2 fluorescence microscope (Oberkochen, Germany) and Axiocam camera (Carl Zeiss, Hertfordshire, UK) in combination with a pE-2 LED excitation system (CoolLED, UK). Excitation LEDs and excitation-emission filter cubes used for the specific fluorophores were: Alexa 488/EGFP – excitation LED 490 nm/filter cube #10 (Zeiss); mCherry/Alexa594 – excitation LED 565 nm/custom filter cube (excitation 560/40 nm, dichroic 585 nm, emission 630/75 nm); DAPI/AMCA – excitation LED 365 nm/filter cube #02 (Zeiss). Fluorescent NAergic neurons were counted in 1:3 serial sections and Abercrombie corrected as previously described (Howorth 2009). All images were initially processed using Zeiss AxioVision 4.7 software (Carl Zeiss, Germany).

Confocal image stacks were captured using a Leica SP5-AOBS confocal laser-scanning microscope. Red (Alexa 594) and green (Alexa 488) fluorescent labeling was visualized using a 2 mW orange HeNe 594 nm laser and a 100 mW Ar laser respectively. Sections were imaged were taken using an oil immersion objective (Leica x63, NA 1.4) at 0.5 um step intervals and captured using Leica software. Parameters were set to optimize z-resolution and a line average of 4 was used to reduce background noise. Stacks were visualized and projected in Volocity 4 (*Improvision*). Figures were prepared for presentation using Adobe Photoshop/Illustrator CS5 for optimization of contrast/brightness and addition of annotation respectively.

### 4.11 Data analysis

All data are presented as mean±standard error of mean (SEM) or median [interquartile range] as appropriate. The normality of data was assessed using the D׳Agostino-Pearson test. Subsequent statistical testing was undertaken using paired and unpaired t-tests, one and two way ANOVA (with Bonferroni׳s post tests), MANOVA (with Tukey׳s post test) and Mann Whitney/Kruskal-Wallis (with Dunn׳s post test) tests as appropriate. Data were analyzed using Prism (Graphpad Prism 5, San Diego, CA, USA) or SPSS (v21, IBM) and differences were considered significant at P<0.05.

## Conflict of interest

The authors declare no competing financial interests

## Figures and Tables

**Table 1:** Pontospinal LC neurons have distinct electrophysiological properties.

	Naïve	LC Injected non transduced	LC Injected transduced	Ps:LC
*N*=	9	14	24	10

Resting Potential (mV)	−58.9±1.0	−59.4±1.3	−58.2±0.6	−59.1±0.8
Firing rate (Hz)	2.2±0.3	2.3±0.3	2.4±0.2	2.3±0.3
Threshold (mV)	−46.1±1.3	−48.1±1.0	−46.4±0.8	−48.7±1.1
AP amplitude (mV)	68.2±1.3	71.8±1.4	70.5±1.2	75.1±2.0
AP duration (ms)	**1.51**±**0.06**^⁎⁎⁎⁎^	**1.66**±**0.05**^⁎⁎⁎⁎^	**1.63**±**0.05**^⁎⁎⁎⁎^	**1.05**±**0.04**
AHP Amplitude (mV)	**−27.8**±**1.2**^⁎⁎⁎^	**−26.4**±**1.1**^⁎⁎^	**−26.8**±**0.7**^⁎⁎⁎^	**−21.3**±**1.0**
AHP Duration (ms)	194±20	177±21	195±15	212±20
Input resistance (MΩ)	222±14	223±9	243±10	216±16
Time constant (ms)	36.2±3.7	35.5±2.3	36.1±2.0	36.7±2.6

Electrophysiological properties for whole cell patch clamp recordings of LC neurons. Direct transduction with CAV2-PRS-ChR2-mCherry was not associated with any change in electrophysiological properties of the LC neurons. The ps:LC neurons have significantly shorter action potentials and smaller AHPs than control LC neurons recorded from the core of the nucleus (both LC transduced/non-transduced or naive). Neurons recorded 7-14 days after vector injection to either LC or to lumbar dorsal horn (P28-35). MANOVA with Tukey post hoc tests.

^⁎⁎^*P*<0.01; ^⁎⁎⁎^*P*<0.001; ^⁎⁎⁎⁎^*P*<0.0001 compared to ps:LC neurons – none of the other across group comparisons were significant.

**Table 2:** Quantitation of retrograde labeling of pontospinal noradrenergic neurons by CAV2-PRS-ChR2-mCherry.

**Cell count in each pontine nucleus**	**Medium titre CAV2 (0.9×10**^**10**^ **TU/ml)**	**High titre CAV2 (1.2×10**^**12**^ **TU/ml=2.6×10**^**12**^ **pp/ml)**
LC	360±115 (75%)	546±80 (74%)
A5	31±10 (7%)	102±66 (14%)
A7	87±48 (18%)	85±26 (12%)
Total in Pons	479±161	733±170

Vector injections (4×500 nl, two per side) made to lumbar dorsal horn in segments L3/4. (Mean±SEM, *n*=3 rats per titre, proportion of the total number of retrogradely labeled NA neurons given per nucleus in brackets).

**Table 3:** Projection patterns of LC neurons retrogradely labeled from lumbar spinal cord and pre-frontal (CG1) areas.

Region	ps:LC	pfc:LC	Double labeled
Prefrontal cortex (CG1)	+	+++	Sporadic
Insular cortex	+	++	None
Piriform cortex	+	++	None
Hippocampus	+/−	+	None
Thalamus	++ (AV thalamus)	+++ (reticular thalamus)	None
Periaqueductal grey	++	+/−	None
Dorsal noradrenergic bundle	+	+++	Sparse
Cerebellum	++	+/−	None
Inferior Olive	++	–	None
Spinal cord	+++	–	Very sporadic

Distribution of axonal projection fibers seen in each region after retrograde transduction of PFC (CAV2-PRS- EGFP-2A-PSAM) and spinal (CAV2-PRS-ChR2-mCherry) LC modules (*n*=3). Projection fibers revealed after immunocytochemistry for mCherry and EGFP respectively. All brainstem and forebrain sections examined contained both mCherry and EGFP positive fibers in ascending fiber tracts like the dorsal noradrenergic bundle or the medial forebrain bundle. Quantification on arbitrary scale: no fibers −; very low density +/−, low density +; Moderate density ++; High density +++. AV – Anteroventral thalamus.
