# Supplementary material for: Retrograde optogenetic characterization of the pontospinal module of the locus coeruleus with a canine adenoviral vector
Source: Brain Res. 2016 Jun 15;1641(Pt B):274–90. doi: 10.1016/j.brainres.2016.02.023 (PMC5282403; doi:10.1016/j.brainres.2016.02.023)
Supplement: Supplementary file 1 — Supplementary material [file mmc1.pdf]

Supplemental figure 1 Optogenetically-induced spike discharge latency is inversely related to the magnitude of ChR2 current.

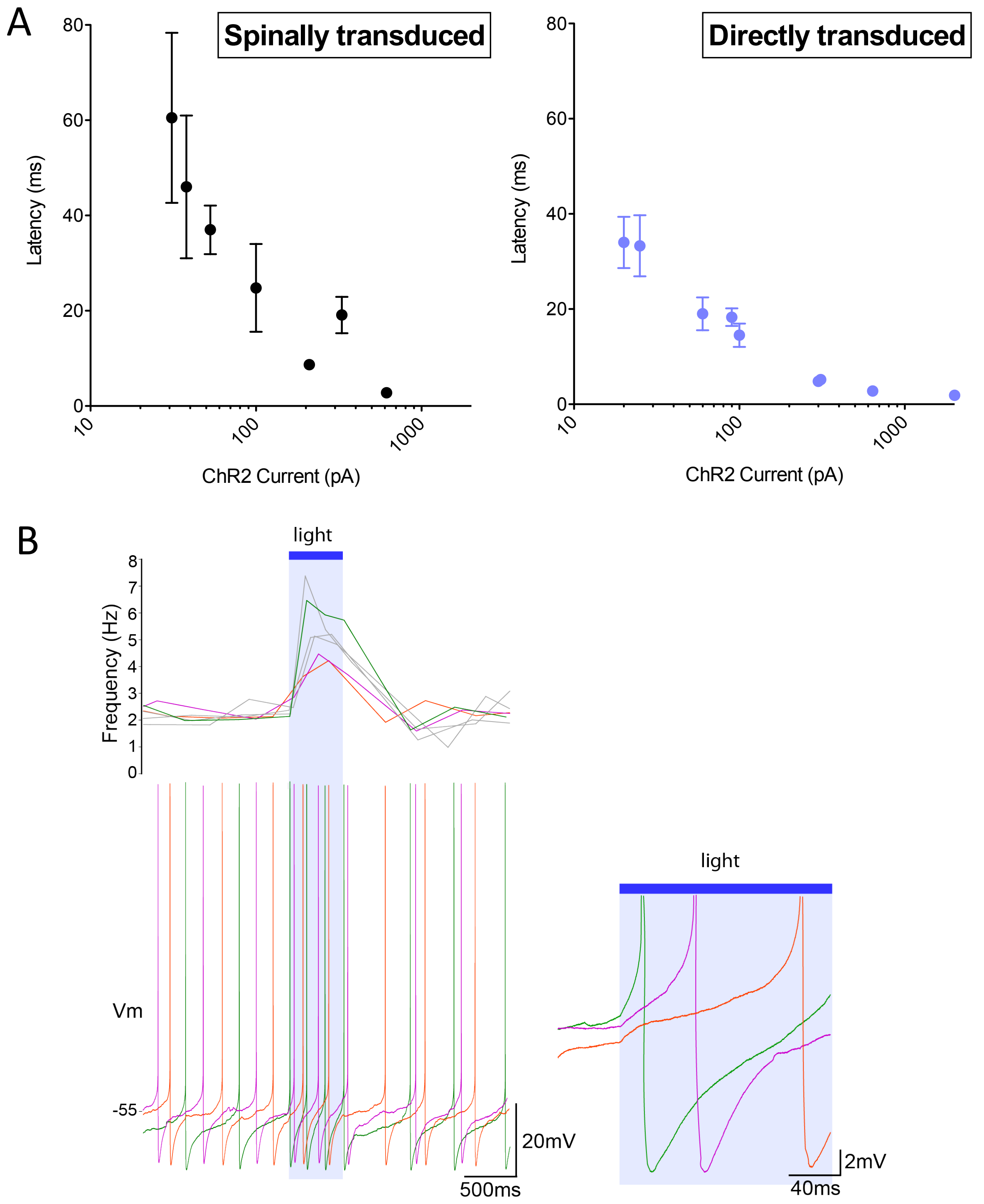

Figure 1 A. Plot of the latency to first action potential across LC neurons evoked by equivalent light pulses (500ms, 10mW, 473nm) versus the magnitude of the ChR2 current (recorded in voltage clamp at -60mV for each neuron). Note the inverse relationship between the magnitude of the current and the latency (and jitter) with optogenetically-induced spikes. Note that for both groups of neurons only a minority reliably fire action potentials response to a light pulse of ≤ 10ms. Cells were either spinally or directly transduced with CAV2-PRS-ChR2-mCherry 7-10 days before subsequent whole cell recordings from identified mCherry fluorescent LC neurons in pontine slices. **B.** Whole cell recording from a pontospinal LC with a small (30pA) ChR2 current to light pulses (500ms, 10mW, 473nm) but nonetheless activation of ChR2 was sufficient to produce a robust increase in cell firing (instantaneous frequency plot for six light flashes shown above and three successive membrane potential responses overlaid below). The latency to spike discharge had a mean value of 60ms with considerable variance as seen in the expanded overlay.
